# Polymeric nanocarriers for the treatment of systemic iron overload

**DOI:** 10.1186/s40591-015-0039-1

**Published:** 2015-03-24

**Authors:** Jasmine L Hamilton, Jayachandran N Kizhakkedathu

**Affiliations:** The Centre for Blood Research, Department of Pathology and Laboratory Medicine, Vancouver, BC V6T 1Z3 Canada; Department of Chemistry, University of British Columbia, 2350 Health Sciences Mall, Vancouver, BC V6T 1Z3 Canada

**Keywords:** Iron overload, Iron chelation therapy, Iron chelators, Desferrioxamine, Deferiprone, Desferasirox, Nanomaterials, Polymeric chelators

## Abstract

Desferrioxamine (DFO), deferiprone (L1) and desferasirox (ICL-670) are clinically approved iron chelators used to treat secondary iron overload. Although iron chelators have been utilized since the 1960s and there has been much improvement in available therapy, there is still the need for new drug candidates due to limited long-term efficacy and drug toxicity. Moreover, all currently approved iron chelators are of low molecular weight (MW) (<600 Da) and the objectives reported for the “ideal” chelator of low MW, including possessing the ability to promote iron excretion without causing toxic side effects, has proven difficult to realize in practice. With prolonged iron chelator use, patients may develop toxicities or become insensitive. In contrast, the limited research that has been geared towards developing higher MW, polymeric, long circulating iron chelators has shown promise. The inherent potential of polymeric iron chelators toward longer plasma half-lives and reduction in toxicity provides optimism and may be a significant addition to the currently available low MW iron chelators. This article reviews knowledge pertaining to this theme, highlights some unique advantages that these nanomedicines have in treating systemic iron overload as well as their potential utility in the treatment of other disease states.

## Review

### Iron

Iron is essential for oxygen transport, DNA synthesis and energy metabolism [1]. Thus, it is life sustaining in virtually all living organisms. The usefulness of iron results from its ability to cycle between its ferrous (Fe^2+^) and ferric (Fe^3+^) forms in oxidation and reduction reactions [1,2]. Although iron is abundant in the earth’s crust, Fe^2+^ is highly toxic, while Fe^3+^ is insoluble in aqueous solution at physiological pH, rendering it inaccessible. As a result, obtaining bioavailable iron is a continual challenge which living organisms have overcome by evolving to conserve iron [1-3]. Organisms have acquired highly organized mechanisms of iron acquisition, transport and storage; microorganisms use low molecular weight (MW) high affinity iron ligands or siderophores, while more complex life forms like mammals use specialized storage and transport proteins [3].

Under normal physiological conditions, the human body contains 3.5-5 g of iron, with the majority (over 70%) existing as hemoglobin [3]. The remainder is found in myoglobin, intracellular storage iron in the hepatocytes of the liver, spleen and bone marrow macrophages, and in proteins and enzymes that are involved in cellular respiration [1-3].

**Figure 1:**
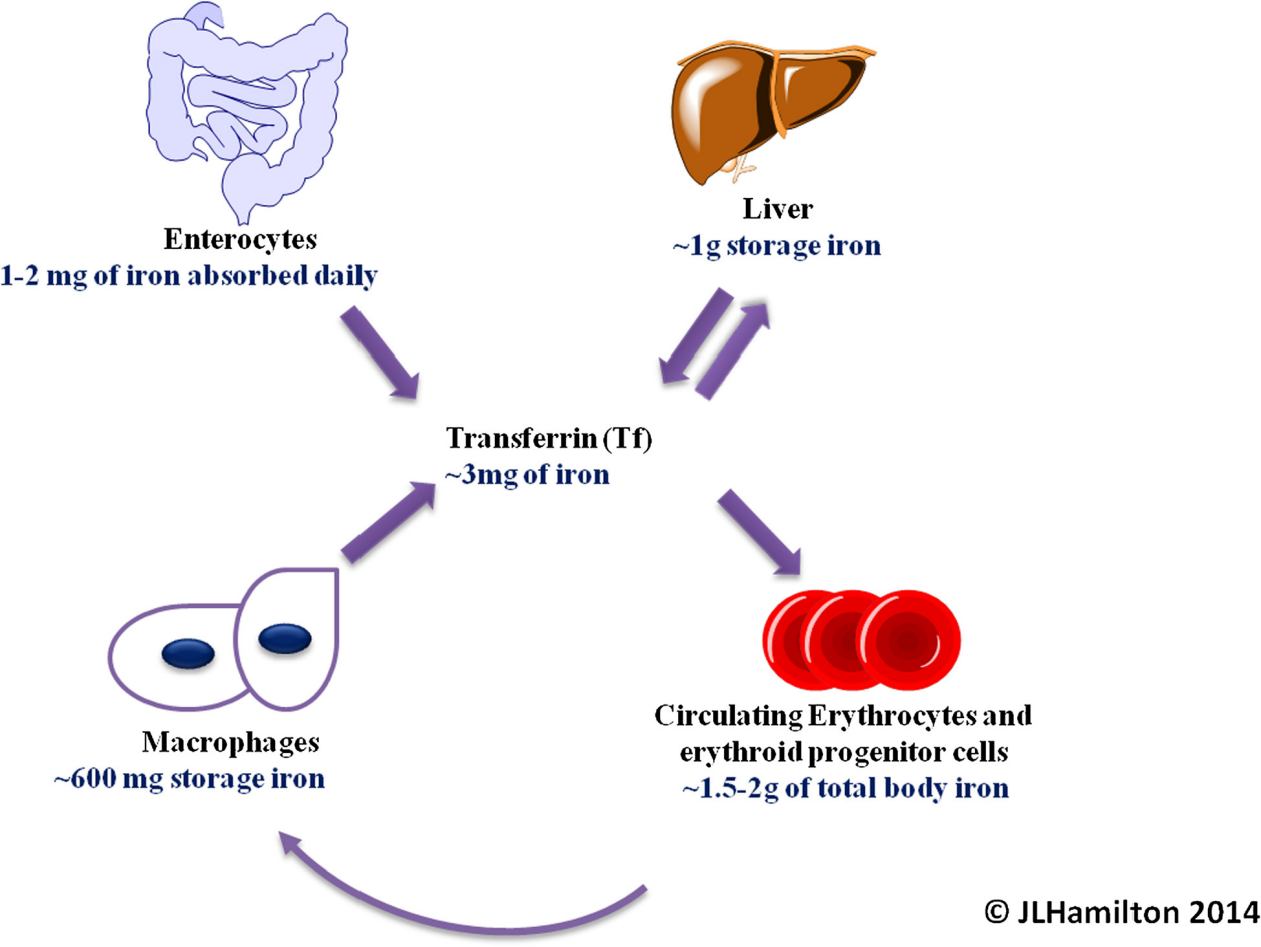
**Iron recycling and distribution in the body.** Body iron is primarily located in erythrocytes (>70%) which are efficiently recycled by macrophages of the liver and spleen. Enterocytes obtain iron from the diet. Macrophages, which obtain iron from the phagocytosis of senescent RBCs release iron into the circulation where it binds to plasma transferrin, the iron transport protein. Transferrin delivers iron to the erythron of the bone marrow and to other sites like hepatocytes of the liver, the main iron storage site in the body. There is no physiological excretion pathway for iron.

Iron metabolism is highly conservative in man, with the efficient recycling of hemoglobin iron forming the major component of iron regulation. Intestinal iron uptake also plays a key role in maintaining human iron homeostasis; 1–2 mg of dietary iron is absorbed daily and approximately 1–2 mg of iron is lost daily due to the sloughing off of epithelial cells, secretions from the skin and gut, and small losses of blood from the gastrointestinal tract [2,3]. This indicates the conservative nature of iron metabolism and recycling. The process of iron recycling and metabolism is schematically demonstrated in Figure 1.

Under normal physiological conditions, iron is complexed with proteins like transferrin (Tf) or other iron binding proteins which ensure that it is unable to cause free radical production [4,5]. In the plasma, iron is transported by Tf and is unavailable for redox activity. Tf has a high iron binding capacity which prohibits the accumulation of toxic unshielded or non-transferrin bound iron (NTBI). Tf contains 2–3 mg of iron and is hypo saturated at ~30% under normal physiological conditions. Tf delivers iron to hepatocytes and specific binding sites on red cell precursors of the bone marrow involved in the synthesis of hemoglobin. Tf also captures iron released into the plasma from intestinal enterocytes or cells which catabolize senescent RBCs [4,5].

Within cells, ferritin is the major storage molecule for reusable iron and accounts for ~27% (1 g) of the total body iron in normal individuals [6]. Ferritin has a storage capacity of 4500 atoms of iron per ferritin molecule and iron storage in ferritin ensures that iron is stored within cells in a safe redox inactive form. Therefore, ferritin reduces the toxicity from free radical generation while ensuring that iron is also available for mobilization for metabolic processes. Ferritin also helps to re-establish normal redox conditions during oxidative stress by removing ferrous ions and oxygen from the cytoplasm. Under pathological conditions in which ‘iron overload’ occur, excess iron is deposited as insoluble ‘iron cores’ of partially degraded ferritin or hemosiderin, primarily in liver, spleen, endocrine organs and myocardium of the heart [1,2].

**Figure 2:**
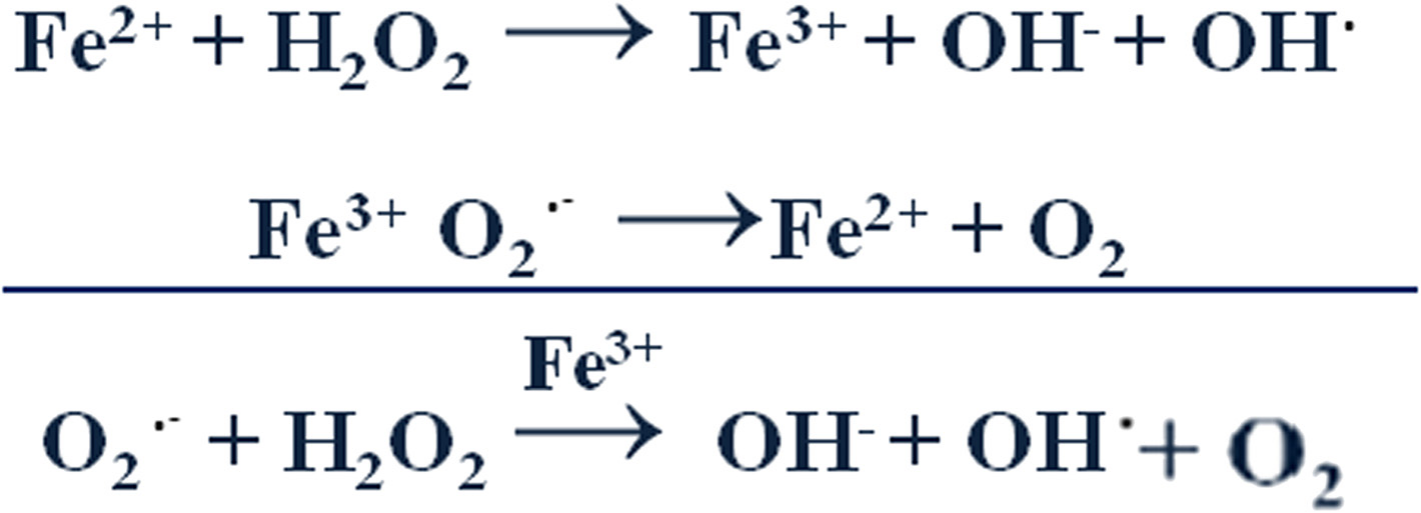
**Harmful redox cycling of iron.** Iron (Fe) can participate in one electron oxidation and reduction reactions. This leads to the generation of harmful free radicals in the presence of oxygen. The hydroxide radical and hydroxide anion (OH^−^) are produced when hydrogen peroxide reacts with ferrous iron. Ferric iron is in turn reduced by the superoxide radical (O_2_·-). Redox active or “labile” iron reacts with cellular hydrogen peroxide (H_2_O_2_) producing the hydroxyl radical (OH·), which perpetuates free radical production, ultimately increasing cellular reactive oxygen species generation. The resulting oxidative stress is associated with damage to cellular components and organs [7].

Although electron shuttling is vital in metabolic processes, under conditions of excess, iron may catalyze harmful reactions that generate free radicals which amplify the development of reactive oxygen species (Figure 2) [7]. This may occur via the Haber-Weiss reaction in which hydrogen peroxide (H_2_O_2_) reacts with the superoxide radical (O_2_) to produce the hydroxyl radical (OH·), the most reactive radical in the body [7]. Although this reaction occurs at minimal levels under normal physiological conditions, it can be catalyzed by iron, leading to accumulation of free radicals, which can interact with cellular components and disturb metabolic functions [7]. It has been shown that the increased generation of free radicals can oxidize lipids, proteins, and DNA in major organs with the heart being most susceptible. Thus, the disruption of normal cellular redox equilibrium is possible with very small amounts of misplaced iron, and the magnitude of the body iron burden is the most important determinant of the ensuing organ damage.

### Transfusion associated iron overload

In contrast to the highly evolved methods of iron acquisition, storage and transport mechanisms, the ability to offload excess iron remains challenging as there is no known physiological pathway to actively excrete iron. This is the major challenge in disease states like β-thalassemia (β-TM), sickle cell disease (SCD) and myelodysplastic syndromes (MDS) that invariably lead to iron overload [1-3,8,9].

Red blood cell (RBC) transfusions are used to ameliorate anemia in patients with β-TM and MDS and can prevent vaso-occlusive events in SCD [8,9]. Patients with these disorders develop severe anemia due to ineffective erythropoiesis and hemolysis, which causes large numbers of marrow erythrocyte precursors to undergo apoptosis before maturity into erythrocytes. This leads to severe anemia. In the case of SCD, there is the added risk of stroke due to the lack of deformability and enhanced stickiness of RBCs which may cause the obstructive adhesion of sickled cells to each other and the vasculature [9].

Further, ineffective erythropoiesis results in a drastic increase in plasma iron turnover, with the turnover of plasma iron occurring at a rate that is 10–15 times greater than in patients with normal erythropoiesis [10]. As a result, patients can accumulate over 2.5 g of iron annually from this process, which results in a “primary” iron overload state. In addition to this inherent iron accumulation, patients receive red blood cell transfusions frequently, which, although highly beneficial in suppressing erythropoiesis and anemia, put patients at risk of developing “secondary” or transfusion associated iron overload.

Each unit of RBC contains approximately 250 mg of iron [8]. Since humans lack an iron excretion pathway, chronically transfused patients accumulate excess iron at a rate of 0.2-0.4 mg/kg/day if transfused more than twice per year [8,10]. This excess iron accumulation causes a saturation of the body’s iron regulatory mechanisms and a subsequent disruption of normal iron regulation.

As the iron loading from transfusions increase, transferrin in the plasma becomes saturated and NTBI appears in the serum [11]. This toxic pool of partially ligated iron accumulates in plasma and is subsequently, and in some cases, preferentially taken up by cells. For example, the rate of NTBI uptake by cultured rat heart cells is greater than 300 times that of transferrin bound iron [10]. As this toxic pool of NTBI accumulates, an intracellular labile iron pool (LIP) is formed and ultimately facilitates harmful redox damage to tissues through the formation of the free hydroxyl radical.

**Figure 3:**
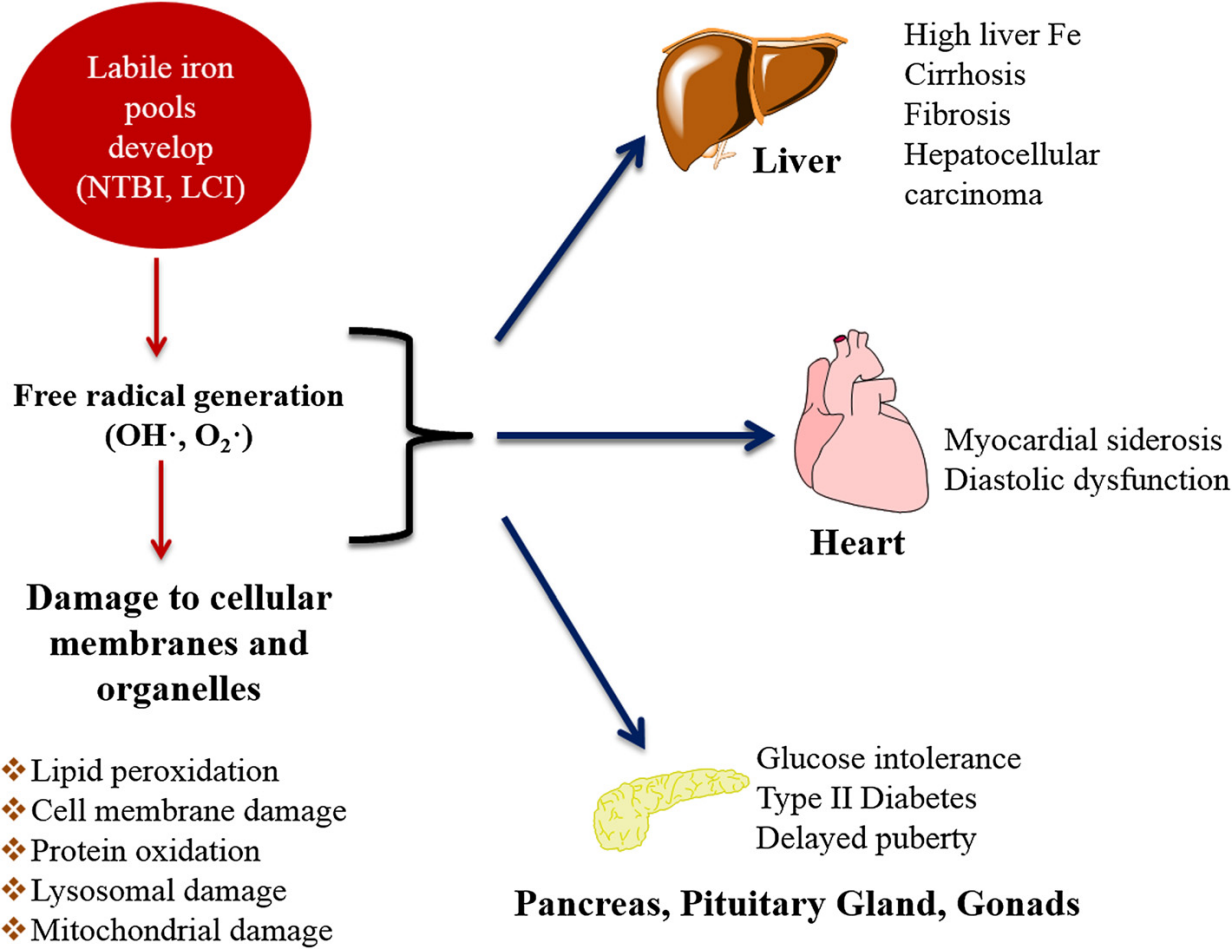
**Excess labile iron causes damage to the body’s organ systems.** Chronic transfusion therapy results in the saturation of serum transferrin and the development of toxic iron pools in cells and tissues. NTBI in the plasma and labile cellular iron (LCI) react with cellular membranes and organelles, causing peroxidation, DNA damage and protein dysfunction. The liver, heart, pancreas and other endocrine organs are most commonly damaged. These events ultimately lead to organ dysfunction, failure and death if left untreated.

The excess iron is accumulated primarily in the liver, spleen, endocrine organs and myocardium. The cytosolic LIP mirrors the cellular iron content and its fluctuations are considered to trigger homeostatic adaptive responses. Once homeostatic mechanisms become saturated, excess iron can ultimately lead to organ dysfunction and death if left untreated [8,10,12]. Figure 3 shows some of the potential effects of iron overload on major organs.

### Iron chelation therapy: treatment of transfusion associated iron overload

**Figure 4:**
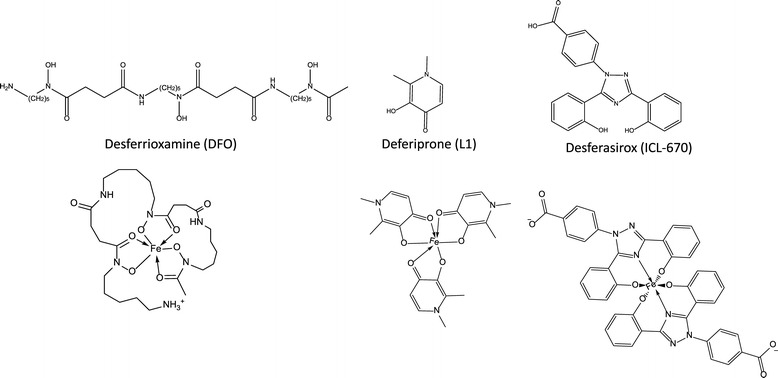
**The chemical structure of clinically approved iron chelators and their iron (III) complexes.**

Iron chelation therapy is clinically indicated for the treatment of transfusion dependent patients with β-TM, SCD and MDS [8,10,13,14]. Iron chelation therapy involves the use of molecules which can bind iron under physiological conditions (iron chelators) to form a non-toxic complex or “chelate” which is subsequently excreted via the feces and/urine, enabling safer body iron levels. Iron chelation therapy protects cells against oxidative damage by reducing the pool of reactive iron in the plasma and cytosolic LIP in cells. Iron chelation therapy inhibits the lipid peroxidation, protein oxidation and cellular damage that accompanies iron overload [15,16]. ICT is recommended after receiving 10–20 transfusions of erythrocytes in order to prevent severe iron loading and damage in major organs [8,10]. Currently, three iron chelators are approved for treating transfusion associated iron overload (Figure 4).

### Desferrioxamine

The most thoroughly characterized iron chelating drug is desferrioxamine (Desferal®, DFO) which has been the standard of therapy for over 40 years. Used since the 1960s, DFO has demonstrated efficacy in prolonging life and improving quality of life for transfusion dependent thalassemic patients (8,13,17). DFO has demonstrated efficacy at preventing lipid peroxidation which leads to organ damage, promoting iron excretion, arresting fibrosis, significantly decreasing deaths by cardiac disease reducing hepatic iron concentrations and extending lifespan in iron overloaded patients [12-14,16,17].

DFO is a high affinity iron (III) chelator with a log stability constant of 30 for the Fe(III) complex and a molecular weight of 560 Da. Due to its hexadentate nature, DFO binds iron in a 1:1 ratio producing a stable complex that prevents iron from producing harmful free radicals [18]. DFO can access iron by two methods; directly interacting with hepatocellular iron and subsequent biliary excretion, as well as from the destruction of RBCs in the reticuloendothelial cells (RES), directly or following its release into plasma as NTBI [8,10]. DFO enters the liver via active transport and interacts with liver and extracellular iron. This leads to excretion primarily by urine as well as some biliary iron excretion [10]. The DFO–Fe(III) complex (Figure 4) does not redox-cycle and this reduces the chances of iron redistribution and toxicity within the body [18].

DFO therapy improves lifespan and quality of life [13,14,17]. Borgna-Pignatti *et al*. showed that mortality at 20 years of age had fallen significantly after the advent of DFO chelation; diabetes had fallen from 15.5% in those born between 1970-74 when DFO therapy was relatively new, to 0.8% in those born after 1980, when DFO was more frequently prescribed [17]. This strongly supports the idea that the age at which transfusion dependent patients begins DFO therapy, as well as adherence to therapy may modulate risk of heart disease and other complications. Similar studies in the UK have shown the benefit of iron chelation with DFO in prolonging life, significantly reducing the incidence of cardiac disease, liver failure and other endocrine disorders in compliant patients [13].

Despite these advantages, DFO is hardly the “ideal” chelator. Due to its low lipophilicity and high MW (560 Da), DFO is not readily absorbed by gastrointestinal cells. In addition, it has a very short circulation half-life of ~20 minutes in humans and must be subcutaneously infused at doses of 40–60 mg/kg for 8–12 h a day, 5–7 days per week. Additionally, DFO at high doses has been associated with severe neurotoxicity, causing sensorineural hearing loss, visual electroretinographic disturbances, and impaired growth and bone development [19-21]. Thus, the use of DFO has been hindered by its shortcomings and attempts toward generating more efficient iron chelators have continued.

### Deferiprone

Deferiprone (Ferriprox®, Cipla, L1) is the second chelator to receive approval for the treatment of iron overload. It was first reported as a potential orally active iron chelator and efficient at *in vivo* iron removal in 1987 and was subsequently licensed for use in India in 1994 and Europe in 1999 with special conditions [22-24]. Due to questions regarding safety and chelation efficiency, L1 only received full marketing authorization in Europe in 2002 and by the FDA in 2011.

L1 is a bidentate chelator thus, 3 L1 molecules are needed to chelate one atom of iron [22,23]. As a result, the efficacy of L1 as an iron chelator is highly dependent on the concentration ratio of chelator and iron in the environment. At low L1 to iron concentrations, L1 may bind to iron incompletely. These partially bound forms of iron with unoccupied coordination sites may accumulate and remain reactive. Furthermore, these partially chelated forms of iron are able to catalyze the formation of harmful radicals and other reactive oxygen species [23].

In the first study reporting its efficacy, L1 was shown to cause iron excretion at a rate proportional to the iron load of the patients and the dose given in the 4 MDS and 4 β-thalassemia major patients participating in the study [22]. Further, the iron excretion levels in urine were found to be similar to that obtained with therapeutic doses of DFO. Rombos *et al.* reported that L1 was safe and caused a reduction in iron overload in Greek thalassemics without causing considerable side effects [24].

However, several subsequent studies have shown that L1 therapy alone may be ineffective in ensuring negative iron balance in many patients, especially in patients with less severe iron loading [25-28]. Hoffbrand *et al*. found no significant reduction in urinary iron excretion in any of the patients enrolled in the study and no significant change in the serum ferritin levels of more than half of the patients that received L1 treatment for more than 3 years [28]. While Cohen *et al*. found that L1 can reduce and maintain body iron in some but not all patients; L1 did not reduce body iron overload to a level below that achieved by DFO in those patients that had lower baseline iron levels [26]. This demonstrates that the daily L1 dose of 75 mg/kg body weight/day induces less iron excretion than the standard daily dose of DFO 50 mg/kg body weight/day.

Other studies show that L1 reduces serum FTN levels in some but not all patients and that the effects of prolonged therapy were not sustained [27]. In addition, although L1 can mobilize iron intracellularly and has been shown to reduce cardiac iron it is unable to promote adequate iron removal and prevent death by cardiac disease in some patients. This was well described in a review by Hoffbrand *et al*. For example, it was reported that 9 out of 532 thalassemic patients undergoing consistent L1 treatment for 3 years died of heart failure [25]. In addition, Hoffbrand *et al*. found that out of 51 L1-treated patients, 4 out of the 5 patient deaths were caused by cardiac dysfunction [28]. This indicates that in some patients with myocardial iron overload and continuing need for blood transfusions, L1 was not reliable at preventing further iron loading [28].

One of the major reasons for the limited efficacy of L1 in clinical use is its rapid metabolism in the liver. The 3-hydroxyl functional group that is found on the L1 molecule is required for effective iron chelation. However, this is also the site of rapid metabolism by glucoronidation in liver cells [18,25,27]. Studies which measured L1 recovery in the urine found that over 85% of the L1 dose given to patients may be recovered in the urine as the inactive 3-O-glucuronide conjugate [18,25].

In addition to the challenging metabolism of L1 described above, severe side effects of L1 can also be limiting without adequate monitoring of patients. Agranulocytosis is considered to be the most serious side effect of L1 use [8,10,26-28]. Milder neutropenia is also common, occurring in up to 4.8% of patients in some studies [25]. Thus, it is necessary to carefully monitor blood counts, especially in patients that are given higher doses. Arthralgia, nausea, gastrointestinal symptoms, zinc deficiency and fluctuating liver enzymes have also been reported [25-28].

### Desferasirox

Desferasirox (Exjade®, ICL-670) is the second orally active iron chelator and the most recent to become approved for the treatment of transfusion associated iron overload [29-32]. It has a MW of 373 Da. Although tridentate, that is, requiring 2 molecules to bind each iron atom, ICL-670 has been shown to be highly selective for iron without promoting the excretion of other metals like zinc and copper [29]. Studies in rats and humans demonstrate that ICL-670 possesses a half-life of 8–16 h, which allows ICL-670 plasma levels to be sustained at a therapeutic range for longer than either DFO or L1. Subsequent clinical studies have confirmed the iron chelation efficacy of ICL-670.

ICL-670 has been reported to be significantly more efficient than DFO and L1 at promoting iron excretion. At equal molar concentrations ICL-670 is reported to be five times more efficient than DFO and ten times more effective than L1 [29]. Several studies show a linear dose-dependent increase in the amount of iron excretion by iron overloaded patients and the doses of ICL-670 given. ICL-670 was reported to induce iron excretion in a manner that would likely prevent iron accumulation in most patients requiring standard transfusion therapy for iron overload [30,31].

Like L1, ICL-670 is highly cell permeable. Moreover, it is absorbed by some cells more rapidly than L1 [32]. The active molecule is highly lipophilic and cell permeable *in vivo* and feces is the main route of excretion for ICL-670 and its metabolites. Renal excretion accounts for approximately 8% [30,31]. Unlike L1, which is absorbed but rapidly inactivated through metabolism, ICL670 rapidly increases in concentration in the plasma of patients and persists at detectable levels for several hours.

Although the long half-life and iron removal efficacy of ICL-670 allows once-daily dosing and offers significant improvement in convenience for patients when compared to DFO and L1, the toxicities reported to accompany prolonged ICL-670 use should be considered [33-37]. In early studies, changes to the renal tubular epithelium were observed as side effects of ICL-670 use [33]. Subsequent studies have confirmed that renal toxicity, hepatic dysfunction and thrombocytopenia are the main concerns for patients undergoing iron chelation therapy with ICL-670. Reports indicate that prolonged use can cause Fanconi syndrome [33-35]. Additionally, a mild, dose-dependent increase in serum creatinine occurs in some patients. Thus, ICL-670 use requires meticulous monitoring of kidney, liver, and hematopoietic function.

In a recent report by Kontoghiorghes, the fatalities associated with ICL-670 use are described to be the highest among the clinically approved chelators. When compared to DFO and L1 which have been in use for much longer periods, the toxicity due to chelation with ICL-670 is high [37]. More importantly, according to this report, ICL-670 was listed as the drug associated with the second highest number of deaths in 2009. Kontoghiorghes, reported that there is a steady increase in the ICL-670 induced deaths in patients per year and that most are caused in elderly patients with MDS. Although the evidence presented in this report was questioned by Riva, the potential seriousness of ICL-670 induced toxicity should not be overlooked [38].

### Continuous advances toward the development of improved iron chelators

The shortcomings of DFO, L1 and ICL-670 highlight the need for improved chelation therapy. Challenges such as the inefficiency of DFO and necessity for continuous subcutaneous infusion; the toxicities of L1 and its inability to adequately control body iron levels with prolonged use; and the severe toxicities associated with ICL-670, have sustained the interest among researchers to develop better options for iron chelation.

**Figure 5:**
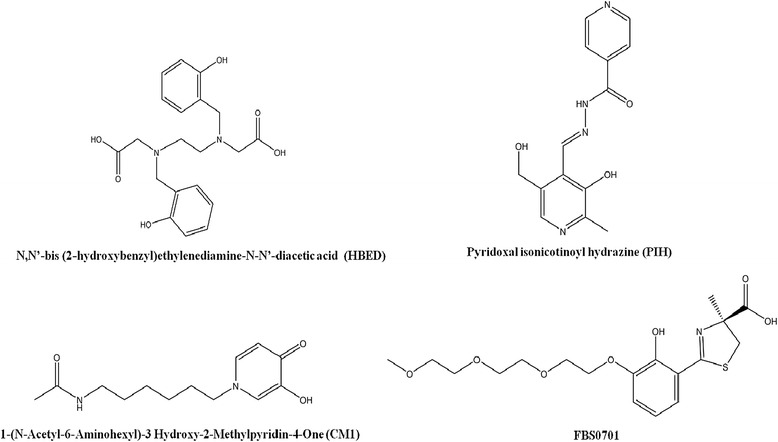
**The structures of previously reported and potential iron chelators for treating secondary iron overload.**

Fe(III) selective chelators are the most fitting for biological applications because they are less likely to deplete other essential metals, which are commonly divalent. However, because the size of a drug influences intestinal absorption, creating orally active hexadentate chelators has proven difficult [39]. Instead, the greatest emphases have been placed on generating novel bi and tridentate chelators or modifying the properties of existing ligands. Over the years, several promising agents which vary in denticity, metal selectivity for Fe(III), toxicity, stability of the Fe-chelator complex and lipophilicity have been proposed and tested in several *in vivo* models including rodents, marmosets, dogs, and primates, with promising agents progressing to clinical trials [40-43]. Figure 5 shows the structures of a few previously reported iron chelators with potential clinical utility.

### HBED

N, N’-bis (2-hydroxybenzyl) ethylenediamine-N-N’-diacetic acid (HBED) is a hexadentate, phenolic aminocarboxylate (MW 388) which has been tested for utility as an iron chelating agent (Figure 5). HBED has a high affinity and specificity for Fe(III) and like DFO, renders it virtually inert and incapable of forming harmful radicals which damage cellular components and organs [40]. HBED has been thoroughly characterized for iron chelation efficiency and toxicity. Initial studies showed that HBED is ineffective at promoting iron excretion when given orally. However, when given subcutaneously or intravenously, HBED was more than twice as effective as DFO at mobilizing iron from rats and cebus apella. Importantly, HBED is a powerful antioxidant and was not associated with any major toxicity in the models tested. Compared to DFO, HBED showed potential as a therapeutic for treating transfusional iron overload with the potential for dosing every other day. HBED has been used in man but has not been further developed for use in treating transfusional iron overload [40].

### Pyridoxal isonicotinoyl hydrazone (PIH)

Pyridoxal isonicotinoyl hydrazone (PIH) is tridentate iron chelator (MW 287) effective at scavenging and mobilizing iron (Figure 5). The potential utility of this class of chelator, as well as its efficacy as anti-proliferative agents, preventing free-radical mediated injury has been documented. [41]. PIH has been shown to chelate both forms of iron, and like other chelators it caused depletion in zinc levels at physiological pH. At neutral pH, the neutral charge of PIH ensures oral absorption and allows access to the cytosol, where labile iron can be chelated. PIH showed efficacy at removing iron from rat reticulocytes which contained labile non-heme iron and mobilizing iron from Chang cells. PIH also reduced iron levels in major organs in mice and was tested in man but did not promote adequate iron excretion. PIH has been reported to be toxic in cebus monkeys and although analogues of PIH have been created none has been further developed for use in treating iron overload in transfusion dependent patients [41].

### FBS0701

FBS0701 (SPD-602) is a novel tridentate chelator of the desazadesferriothiocin (DADFT) class and has been tested in phase II clinical trials (Figure 5). FBS0701 has a MW of 400 (salt form 440), binds iron tightly and has a higher affinity for Fe(III) than other divalent metals. It can enter cells and has demonstrated efficacy in iron chelation and a comparable safety profile to currently approved chelators [42].

A one-week, dose escalation, phase Ib study demonstrated its potential clinical utility and efficacy. While a phase II multicenter trial, which dosed patients with 50–375 mg of the FBS0701-salt, showed a statistical significant reduction in liver iron, confirming the potential benefit of this agent to reduce iron burden from transfusions. Although adverse events were reported, they did not appear to be dose related and occurred at low frequency. Future studies with larger sample sizes will provide more information on the potential of this chelator for treating transfusion associated iron overload. FBS0701 is currently undergoing development and represents a promising agent for future treatment [42].

### CM1

CM1, 1-(N-Acetyl-6-Aminohexyl)-3 Hydroxy-2-Methylpyridin-4-One), is an orally active, bidentate L1 analogue, possessing a MW of 256 Da and is currently being developed for the treatment of iron overload (Figure 5). CMI has shown higher lipophilicity than L1 and can bind both Fe(II) and Fe(III). CM1 is effective at mobilizing cytosolic labile iron in primary mouse hepatocytes and HepG2 cells, and plasma NTBI. It has been studied in transgenic β-thalassemic mice, and has demonstrated efficacy and low toxicity in the liver and peripheral blood of iron overloaded mice. Importantly, CM1 showed efficacy in preventing lipid peroxidation, the underlying cause of cellular damage. Future studies are needed to determine clinical utility of this agent [43].

### Current challenges in iron chelation therapy

#### Monotherapy is inadequate to ensure negative iron balance

Although there are three iron chelators used for the treatment of iron overload, often treatment with any of these chelators alone is not sufficient and it is estimated that 20% of patients undergoing iron chelation therapy will be inadequately chelated [8]. This is due to factors such as poor compliance, inefficacy and toxic side effects. For example, 80% of patients undergoing DFO therapy will experience reactions at the infusion site; oral DFP therapy alone will ensure a negative iron balance in some but not all patients and DFX may cause kidney failure in some patients.

Additionally the safety of iron chelators for certain populations is yet to be clearly defined and remains somewhat controversial. This is especially true for pregnant women. Yet, the increasing lifespan of women to childbearing age and the improvement in therapy over the last few decades has demonstrated the increase in the likelihood and cases of pregnancies in transfusion dependent women and the importance to advance knowledge and treatment options for this vulnerable group of patients.

#### Properties of effective iron chelators

Effective iron chelation therapy is achieved only if iron chelators can remove equal or greater amount amounts of iron to that accumulated due to transfusion therapy. This requires chelators to be able to reach the target sites at relevant concentrations. Since there are several iron pools that develop in iron overload, chelators which are effective at mobilizing iron from all labile iron pools would be advantageous. Secondly, the effective protection of the heart by chelation therapy is critical for iron overloaded patients as heart failure is the leading cause of the death in thalassemia major patients with iron overload [8,10].

An “ideal” chelator should have an ability to bind NTBI over long periods of time in order to ensure adequate coverage. A long acting chelator would ultimately decrease the amount of iron that is taken up into tissues and would prevent harmful, iron-catalyzed reactions. In addition, iron chelators that are clinically effective must have high selectivity for Fe(III) in comparison to other important trace metal ions in the body. Table 1 compares the features of currently available iron chelators with that of “ideal” iron chelator features.

**Table 1:** **Contrasting features of ideal and currently approved iron chelators**

**Chelator property**	**Ideal chelator**	**DFO**	**L1**	**DFX**
Cost	Affordable for patients in low income countries	Moderate	Moderate	Unaffordable and unavailable for most
Route of Administration	Oral	i.v injection or s.c infusion	Oral	Oral
Circulation t_1/2_	Long enough to allow once-daily dosing and effective iron removal	Short (~20 min) requires all-day (8-12 h) delivery	Moderate; requires at least 3 times per day dosing	Ideal; 8–16 hours, requiring once-daily dosing
Therapeutic index	High	High at high doses in patients with high burden	Unpredictable	High
Toxicity	None	Neurotoxic, swelling at infusion sites, bone deformities	Agranulocytosis and mild neutropenia are common	Reversible kidney failure
Unsaturated Iron Binding Capacity	High: Long enough to prevent drastic fluctuations in LIP	None	Moderate	High
Ability to remove iron from heart, liver etc.	High	Low	High	High

#### Overcoming the challenges in current iron chelation therapy: the development of new polymeric iron chelators

**Figure 6:**
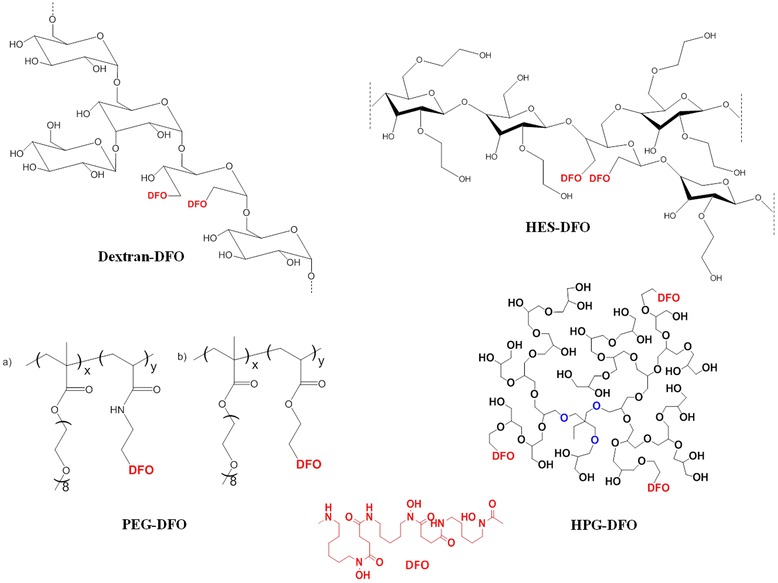
**The chemical structure of polymer components used in DFO modification.**

**Figure 7:**
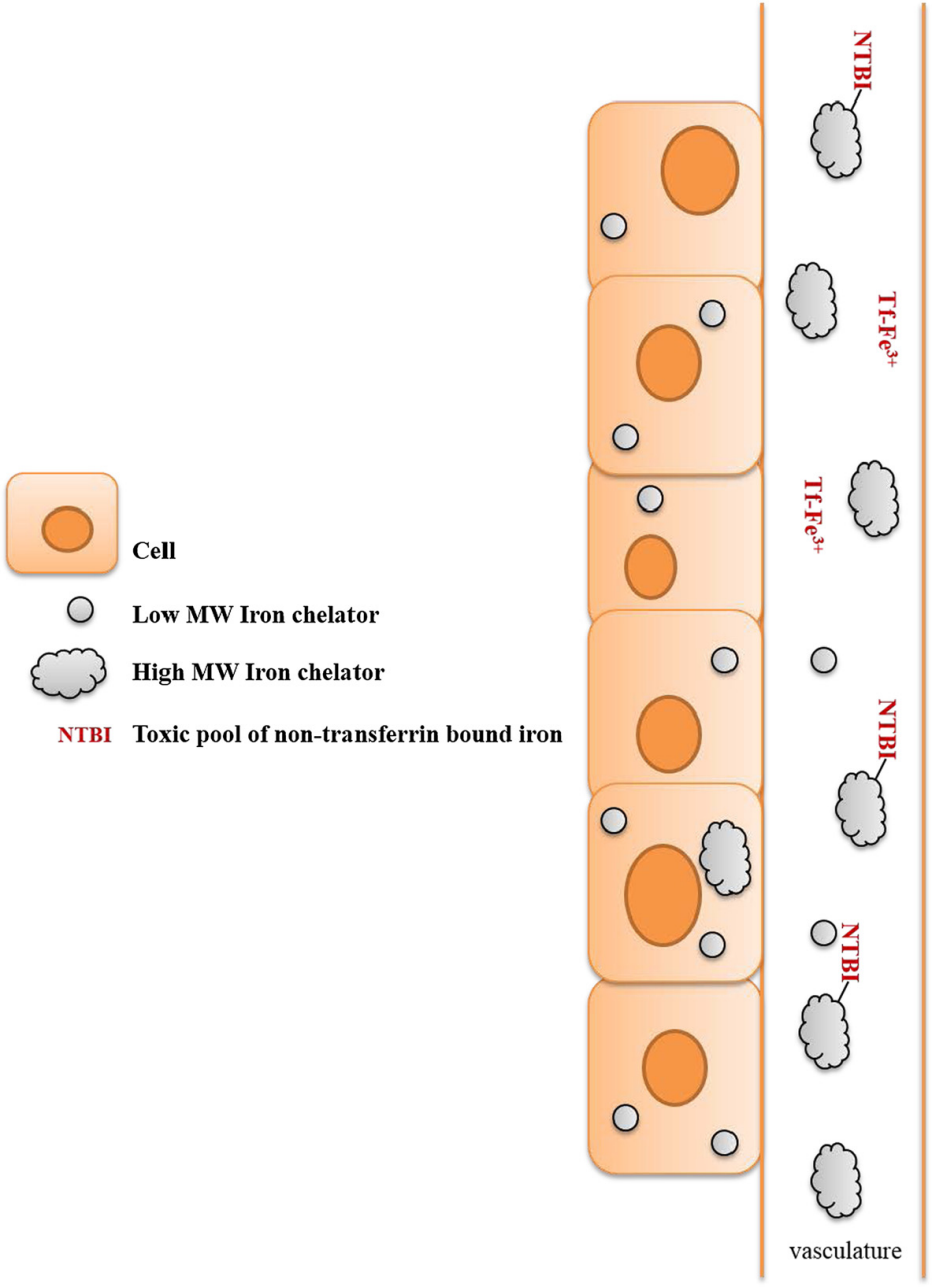
**Polymeric iron chelators result in a higher unsaturated iron binding capacity (UIBC).** High MW chelators have longer half-lives and are not readily taken up by cells. In contrast, small MW chelators are highly permeable to cells and may be rapidly metabolized and removed from the circulation.

Although all currently approved chelators are of low MW, previous reports of polymeric iron chelators have demonstrated that high MW chelators can be a viable alternative for improving the pharmacokinetics and systemic toxicity of small MW chelators (Table 2). The approach to develop polymeric chelators or polymeric nanocarriers for iron chelators has varied from using iron binding dendrimers, hydrogels, the covalent attachment of DFO to a wide range of biocompatible materials and the use of amino acid amide derivatives. Figure 6 shows the structures of polymeric components that have been used to modify DFO toxicity and systemic circulation. The major motivations for producing polymeric iron chelator is to overcome the challenges of rapid plasma elimination, prolonged infusions and widespread DFO toxicity that has hindered the achievement of safe iron levels in many iron overloaded patients undergoing iron chelation therapy Figure 7. Indeed, several studies have indicated that polymeric iron chelators possess unique advantages over their low MW counterparts.

**Table 2:** **The influence of polymer conjugation on the pharmacokinetics and pharmacodynamics of DFO**

**-DFO Conjugate**	**Pharmacodynamic effect**	**Pharmacokinetic effect**
Dextran-DFO [44]	LD50 increased from 250 mg/kg to 4000 mg/kg, reduction of pulmonary hypotension in dogs	Increased circulation half-life
Starch-DFO (40SDO2) [47]	Reduced retinal toxicity in albino rats, Reduction of pulmonary hypotension in dogs	Increased *in vivo* iron excretion efficiency, increased circulation half-life. Excess free iron binding capacity in healthy males
PEG-Methacrylate-DFO [46]	Reduced endothelial cell toxicity	--
HPG-DFO [48]	Increased *in vivo* LD50	Increased *in vivo* iron mobilization efficiency in mice Increased circulation half-life in mice
		Decreased clearance

Conjugated forms of DFO are associated with reduced toxicity both *in vitro* and *in vivo*. Dextran-DFO, HES-DFO and HPG-DFO demonstrated significantly higher plasma half-lives than unconjugated DFO [44,46-48].

In a 1989 report, Hallaway *et al.* described the advantages associated with attaching DFO covalently to dextran and hydroxyethyl starch (HES) [44]. This resulted in a significant increase in the plasma half-life and reduction in toxicity of DFO with no apparent loss in the iron chelating properties. The increase in size of starch conjugated DFO (S-DFO) resulted in improved plasma half-life from 5 min for DFO to 87 min S-DFO in mice. The LD_50_ in mice increased to 4000 mg/kg for dextran-DFO in comparison to 250 mg/kg for DFO and there was an absence of pulmonary hypotension when intravenously administered in dogs. This is a major improvement as DFO and its iron complex induced hypotension in dogs at a dose of 100 mg/kg. Moreover, the blood pressure did not return to normal during the 60 minutes of the experiment. In contrast, neither HES nor dextran caused any significant change in blood pressure. Additionally, the half-life was more than 10 times greater after conjugation.

In 2005 Polomoscanik *et al.* reported the generation of a non-toxic formulation of DFO hydroxamic acid based iron chelating hydrogels and evaluated utility to prevent iron absorption in the gut [45]. These gels were effective in preventing gastric iron absorption and did not cause any change in hemoglobin and hematocrit. They also conducted a study to determine whether other divalent metals compete with Fe(II) for binding to the polymeric chelator and found that Zn and Cu did compete but that overall the binding strength of the polymeric chelators was affected only modestly. These agents prevented the rise of hematocrit and hemoglobin in treated mice and suggest that arresting the intestinal uptake of dietary iron is a viable option for depleting iron levels. The hydrophilic polymeric hydroxamic acid gel was non-toxic and suggests the feasibility of using non-absorbed iron binding polymers as oral agents to sequester dietary iron in the GI tract.

In 2009, our group reported the development of well-defined, blood compatible and degradable PEG based copolymers conjugated with DFO (P-DFO) for application in iron chelation therapy [46]. PEG methacrylate was copolymerized by the RAFT method with a functional monomer for the conjugation of DFO to the polymer backbone via degradable or non-degradable linkage. The presence of PEG increased the biocompatibility of these nanoconjugates. The presence of the hydrolysable ester linkages was anticipated to cause slow degradation of the conjugate via the ester linkages between the PEG side chains and copolymer backbone. P-DFO had MWs ranging from 27–127 kDa with between 5–26 DFO units per polymer chain and demonstrated improved biocompatibility and toxicity profile as compared to unconjugated, small MW DFO. Like dextran and HES-DFO, P-DFO had a drastically improved toxicity profile; while unconjugated DFO exposure in HUVECs resulted in death at 3 μM, P-DFO was associated with ~90% cell viability up to 700 μM. However, to date P-DFO has not been tested for efficacy *in vivo.*

The most significant evidence existing for the potential clinical utility of polymeric iron chelators was published by Harmatz *et al.* who tested a DFO-starch conjugate [47]. In this first reported human clinical trial of polymeric chelators, S-DFO caused clinically significant iron excretion after single dose infusion of S-DFO. Maximum plasma chelator levels of 6 mM/L were achieved by S-DFO after 4 h intravenous infusion, an order of magnitude higher than that which occurs with DFO treatment. More importantly, there was also residual iron binding capacity present in the plasma of patients for one week, without any observable toxicity [47].

Recently, we have achieved the longest half-life in mice recorded to date by conjugating DFO to hyperbranched polyglycerols (HPG) [48]. HPG is a class of versatile, biocompatible, inert, nano polymers that can be synthesized in a controlled one-step reaction with low polydispersity. Detailed biocompatibility testing of these polymers conducted *in vitro* and *in vivo* has demonstrated the unique advantages that HPG may have in nanomedicine [49,50]. Our group has previously developed HPGs as a synthetic substitute for serum albumin that closely mimics the binding and transport properties of natural albumin and is considered to hold advantages over the current clinically used plasma expanders [50]. We have also coated HPG on red blood cells to mask different antigens towards the development of universal blood cells, developed DNA delivery agents, and developed anticoagulant neutralizing agents for use as heparin antidotes [51,52]. Due to the biocompatibility, multi functionality and long circulating nature of high MW HPG, we anticipated that it would be a promising candidate for the development of a new generation of non-toxic macromolecular nanoconjugates for the removal of iron *in vivo*.

HPG based polymeric chelators were developed by conjugating DFO to different MW HPGs with different DFO density, producing a library of polymeric-DFO conjugates, referred herein as HPG-DFO. HPG-DFO conjugates varied in properties depending on their MW and DFO density, and the structural features of HPG-DFO were optimized to achieve long plasma circulation time, high chelation efficiency and low toxicity. All of the HPG-DFO conjugates demonstrated suitable biocompatibility and the hydrodynamic radius ranged from 4.2 to 7.9 nm. The narrow polydispersity of the polymer scaffold allowed the development of homogeneous conjugates with well-defined and predictable characteristics *in vitro* and *in vivo*. The plasma circulation half-life of DFO was increased more than 484-fold (44 h) for a 500 kDa conjugate compared to that of unconjugated DFO. These conjugates were also more efficient at mobilizing iron in mice; the iron excretion was significantly higher in mice treated with HPG-DFO [48]. Ongoing studies which are aimed at understanding the circulation behavior of these molecules with respect to its MW and DFO density will allow further optimization of toxicity and biodistribution.

Apart from the previously described polymeric structures with specific chelators attached to the polymer backbone, other polymeric chelators have also been generated. Winston *et al.* prepared polymeric chelators with hydroxamic acid terminated side chains [53]. These polymeric chelators were composed of amino acid amide derivatives of acrylic and methacrylic acid with the terminal carboxyl group converted to the hydroxamic acid. Polymeric chelators demonstrated a high affinity for iron (III) and were able to remove iron from iron overloaded mice when administered via i.p. injection.

Zhou *et al.* reported the synthesis of 3-hydroxypyridin-4-one hexadentate, ligand-containing copolymers by the copolymerization of 3-hydroxypyridin-4-one hexadentate ligand with *N,N*-dimethylacrylamide (DMAA), and *N,N’*-ethylene-bis-acrylamide (EBAA) using (NH4)_2_S_2_O_8_ as the initiator [54]. This class of chelator has demonstrated high selectivity and affinity for iron (III), and has potential clinical utility for the treatment of iron overload diseases associated with the hyper-absorption of iron (e.g. hemochromatosis).

Since iron accumulates as a result of transfusions as well as dietary absorption, it has been suggested that blocking the intestinal absorption of iron may also significantly reduce iron levels in patients. This has been attempted by administering high affinity, high MW chelators that are not absorbed by intestinal cells, which bind iron and promote its removal from the body. Zhou *et al.* designed hydroxypyridinone-containing polymers which significantly reduced intestinal iron uptake. In their in vitro intestinal perfusion study, the accumulated absorbed iron was significantly reduced compared with the control groups in the presence of polymeric iron chelator.

Dendrimers have also demonstrated suitability as for the generation of polymeric iron chelators [55]. Zhou *et al.* designed novel dendritic iron chelators by terminating dendrimers with hexadentate ligands formed from hydroxypyridinone, hydroxypyranone, and catechol moieties and have demonstrated that these novel conjugates can reduce iron absorption efficiently. This supports the idea that polymeric and dendritic iron chelators may be able to uniquely diminish iron absorption through the intestine and may have clear potential clinical utility due to their high MW [55].

Although the encapsulation of DFO into liposomes has also been attempted as a means of improving the therapeutic index, it has been unsuccessful [56]. This is likely because DFO encapsulation does sequesters DFO only initially, however, release profiles may not be ideal due to the hydrophilicity and relatively large molecular weight. Another reason may be that if untargeted to specific tissues, DFO can still cause toxicity once released into healthy, non-iron overloaded cells. Liposome technology and DFO can be extremely valuable with a targeted approach to a specific organ affected by the iron overload. It may also be much more useful in treating cancers or tumors that have a high iron requirement but it also has to be targeted to ensure a very specific release location. Liposomal encapsulation may also be beneficial if liposomes can be tuned to release DFO slowly. Slow release would be beneficial and would reduce toxicity associated with high DFO doses.

#### Advantages of polymeric iron chelators

There are several advantages associated with the modification of iron chelators with polymeric nanocarriers (Table 2). One of the most important properties of an iron chelator is its circulation half-life as this influences the unsaturated iron binding capacity (UIBC) of the chelator and ultimately, the rate of NTBI generation and removal. As iron in β-TM patients is constantly being turned over due to the RBC catabolism in macrophages or the breakdown of ferritin within cells, these pools of iron are redox active and are mainly responsible for the iron loading of plasma and tissues. Thus, in order to achieve effective iron chelation and the removal of labile iron, 24 h chelation coverage is the ideal. The importance of having a long-circulating chelator and constant coverage has also been demonstrated in studies that have shown that prior to significant changes in cardiac iron in patients, cardiac failure is reversed during continuous administration of DFO and that NTBI appears within minutes of a chelator being cleared from the body [57].

Increased half-life is anticipated to have profound effects on compliance to therapy for patients treated with DFO. The current arduous DFO regimen has proven challenging for many patients, especially young children and teenagers, so an optimized version of a long circulating DFO would be of significant benefit. If polymeric chelators can be engineered toward slow, sustained degradation in the plasma, it is conceivable that patients will require once-weekly or bi-weekly injections that will enable sufficient iron mobilization. In addition to improving compliance, this will likely improve access to DFO for many patients since less drug will be needed to cause effective iron excretion and will reduce toxicities, ultimately allowing a paradigm shift in chelation therapy with DFO.

#### Reduction in toxicity

The high MW polymeric DFO conjugates have demonstrated efficacy at clearing excess iron *in vivo* with reduced or absent toxic effects [44-48]. The reduction in the acute toxicity of polymer conjugated DFO as compared to the parent drug, allows the administration of much greater amounts of “active ingredient” after polymer conjugation. This reduction in toxicity is most likely attributable to a size dependent reduction in cellular uptake as conjugates remain in the vascular space longer once conjugated to polymers. It is well documented that low MW iron chelators can be taken up by many cell types. DFO has the highest MW among the 3 clinically approved chelators and it has the highest hydrophilicity (with a distribution coefficient of −2) at physiological pH. DFO enters the liver via active transport and can interact with the LIP and facilitate the iron excretion [10].

Although a cell permeable chelator is more likely to have access to labile cellular iron, it is not necessary for all chelators to have this property. In fact, in some instances it may be disadvantageous as small chelators which are not specific for Fe(III) or which have high affinities for Fe(II) may chelate other essential metals, thus exerting unwanted effects. This is well demonstrated when considering some of the factors underlying the toxicity of low MW iron chelators. Low MW chelators may remove or displace essential iron or other metals. Iron chelators can interfere with zinc, copper and other micronutrient even though the binding affinity for these metal ions is relatively small (for instance, the log cumulative stability constant of DFO-Fe is 30.6 versus 11.1 for DFO-Zn ^2+^) [18]. Zinc deficiency has been reported in patients undergoing DFO and L1 therapy [58]. Additionally, reducing essential iron in the cell can result in reduced cell proliferation by inhibiting intracellular ribonucleotide reductase and cell division [59].

#### Polymeric iron chelators: beyond transfusional iron overload

Iron is an essential element for several metabolic processes and the perturbation of iron recycling and metabolism has been shown to be a major factor in several diseases. Iron removal has been shown to be a useful approach for the treatment of microbial infectious diseases, reducing the growth rate of some cancer cells and neurodegenerative diseases [60-62]. Additionally, iron chelation may be useful in malaria treatment and treatment of the HIV virus [63,64]. Therefore, the ability to modify polymers to enhance iron chelation, minimize toxicity and maximize blood circulation may prove beneficial in depleting iron stores in these disease states as well. Polymeric iron chelators may also be modified to enhance targeting to specific areas of the body and may thus have potential clinical utility beyond the treatment of iron overload.

For example, polymeric chelators may be uniquely suited for iron depletion at the site of tumors owing to their large size that can be used to passively target tumors through their compromised endothelial junctions via the enhanced permeation and retention (EPR) effect [65]. Likewise, the increased residence time associated with polymeric iron chelators may have utility in treating chemotherapy patients [61]. It has been reported that cancer chemotherapy increases the levels of NTBI due to toxicity of anticancer drugs to bone marrow cells. This can reduce the demand for iron by marrow cells and cause transferrin to become fully loaded which increases NTBI and may render the host more susceptible to oxidative damage. While low MW chelators are prone to enter cells, high MW drugs are almost exclusively restricted to the vascular and extracellular spaces due to the poor cellular uptake. Thus, polymeric chelators are advantageous for such treatments.

## Conclusions

Current iron chelation therapy requires the daily administration of virtually the maximum tolerated doses of DFO, L1 and ICL-670 in order to ensure that the rates of transfusional iron loading and iron excretion in transfusion dependent patients are well matched. This result in patients experiencing a wide range of toxicities and in many cases, the administered doses are still insufficient to mobilize the required amount of iron and produce negative iron balance. As a result, chelators that are less toxic and more efficient at iron mobilization would ensure rapid reduction of labile body iron and prevent the development or progression of complications associated with iron overload. One potentially promising approach to advancing chelation therapy is through the use of polymeric chelators.

The role of polymers in medical applications has seen substantial growth over the past three decades. The use of polymers for applications in drug delivery, for artificial organs, medical devices and dentistry is well documented. In this review we have highlighted the potential of polymeric iron chelators for the removal of toxic iron pools *in vivo*. Through appropriate design and modification of biocompatible polymers, several high MW chelators have been developed and characterized. These conjugates take advantage of the biophysical properties of the polymers that can extend plasma circulation and reduce dose dependent toxicity, while retaining excellent chelating properties. In many aspects, polymeric chelators are shown to be significantly more effective than small MW chelators at *in vivo* iron removal due to extended plasma circulation times and may represent a new paradigm in treating transfusion associated iron overload. Although none of the previously designed polymeric chelators have advanced to the clinic, the successful phase Ib clinical trials conducted Harmatz *et al.* suggest the potential for iron removal that exists when DFO is engineered into a high MW polymer conjugate.

Our experience with modifying the properties of low MW iron chelators like DFO through nanoengineering with polymers has grown only moderately from the first attempt in 1989. It is well established that the undesirable properties of DFO can be significantly transformed by modification with nanomaterials, with such improvements varying based on the properties of the biomaterials used. To date, emphasis has been placed on modifying DFO properties, although the possibility of engineering other small MW iron chelators like L1 and ICL-670 to improve their toxicity profile remains uncertain due to their bi and tri-denticity respectively.

It is essential that polymer components are safe to use, can be reproduced easily on large scale and are suitable for transformation into effective pharmaceutical formulations that are practical to use clinically. Therefore, future studies must consider several important questions. It is important to determine whether polymers such as HPG can be further modified to generate more targeted high MW chelators. It is also important to determine whether the high MW chelators have specific degradation routes or are prone to accumulation with chronic use. Since the MW of drug molecules can play a critical role on their cellular and tissue accumulation, determining the toxicity profile of polymers and their likelihood of reaching target sites in adequate concentrations will be of great importance.
